# 中国埃克替尼治疗非小细胞肺癌专家共识(2015版)

**DOI:** 10.3779/j.issn.1009-3419.2015.07.01

**Published:** 2015-07-20

**Authors:** 远凯 石, 燕 孙, 翠敏 丁, 子平 王, 长利 王, 正 王, 冲 白, 春学 白, 继锋 冯, 晓晴 刘, 方 李, 跃 杨, 永前 束, 密璐 吴, 建行 何, 沂平 张, 树才 张, 公琰 陈, 红鹤 罗, 荣城 罗, 彩存 周, 燕斌 周, 青松 庞, 宏 赵, 琼 赵, 爱琴 顾, 扬 凌, 诚 黄, 宝惠 韩, 顺昌 焦, 红 简

**Affiliations:** 1 100021 北京，中国医学科学院北京协和医学院肿瘤医院，抗肿瘤分子靶向药物临床研究北京市重点实验室 Cancer Hospital, Chinese Academy of Medical Sciences & Peking Union Medical College, Beijing Key Laboratory of Clinical Study on Anticancer Molecular Targeted Drugs, 100021 Beijing, China; 2 050000 石家庄，河北医科大学第四医院 Fourth Hospital of Hebei Medical University, 050000 Shijiazhuang, China; 3 300070 天津，天津医科大学肿瘤医院 Tianjin Medical University Cancer Institute & Hospital, 300070 Tianjin, China; 4 518020 深圳，深圳市人民医院 Shenzhen People' s Hospital, 518020 Shenzhen, China; 5 200433 上海，第二军医大学长海医院 The Second Military Medical University Changhai Hospital, 200433 Shanghai, China; 6 200032 上海，复旦大学附属中山医院 Fudan University Zhongshan Hospital, 200032 Shanghai, China; 7 210009 南京，江苏省肿瘤医院 Jiangsu Cancer Hospital, 210009 Nanjing, China; 8 100071 北京，解放军第307医院 The 307th Hospital of Chinese People's Liberation Army, 100071 Beijing, China; 9 100853 北京，解放军总医院 Chinese People's Liberation Army General Hospital, 100853 Beijing, China; 10 100142 北京，北京肿瘤医院 Beijing Cancer Hospital, 100142 Beijing, China; 11 210029 南京，江苏省人民医院 Jiangsu Province Hospital, 210029 Nanjing, China; 12 810000 西宁，青海大学附属医院 Qinghai University Affiliated Hospital, 810000 Xining, China; 13 510000 广州，广州医科大学附属第一医院 The First Affiliated Hospital of Guangzhou Medical Unversity, 510000 Guangzhou, China; 14 310022 杭州，浙江省肿瘤医院 Zhejiang Cancer Hospital, 310022 Hangzhou, China; 15 101149 北京，首都医科大学附属北京市胸科医院 Beijing Chest Hospital, Capital Medical University, 101149 Beijing, China; 16 150081 哈尔滨，哈尔滨医科大学附属肿瘤医院 Harbin Medical University Cancer Hospital, 150081 Harbin, China; 17 510080 广州，中山大学附属第一医院 The First Affiliated Hospital, Sun Yat-sen University, 510080 Guangzhou, China; 18 510515 广州，南方医科大学南方医院 Nanfang Medical University Nanfang Hospital, 510515 Guangzhou, China; 19 200433 上海，同济大学附属上海市肺科医院 Tongji University Affiliated Shanghai Pulmonary Hospital, 200433 Shanghai, China; 20 310003 杭州，浙江大学附属第一医院 The First Affiliated Hospital, Zhejiang University, 310003 Hangzhou, China; 21 200030 上海，上海交通大学附属胸科医院 Shanghai Chest Hospital, Shanghai Jiaotong University, 200030 Shanghai, China; 22 213001 常州，常州市第四人民医院 Changzhou Fourth People's Hospital, 213001 Changzhou, China; 23 350014 福州，福建省肿瘤医院 Fujian Cancer Hospital, 350014 Fuzhou, China

据《中国2011年恶性肿瘤登记年报》报告，2011年我国肺癌发病率为48.32/10万，死亡率为39.27/10万。发病率和死亡率均居恶性肿瘤的首位^[1]^。非小细胞肺癌(non-small cell lung cancer, NSCLC)占肺癌患者的85%左右，大多数就诊时已属晚期，失去了手术治疗的机会。化疗是晚期NSCLC治疗的主要手段，其地位虽然没有发生根本改变，但其疗效已达到平台，同时化疗的毒副反应也限制了其广泛的临床应用。近年来，由于表皮生长因子受体酪氨酸激酶抑制剂(epidermal growth factor receptor-tyrosine kinase inhibitor, EGFR-TKI)确切的疗效、轻微的不良反应和口服给药的便利等特点，突破了传统化疗药物的瓶颈，已经成为晚期NSCLC治疗中不可或缺的重要手段。目前我国已上市的EGFR-TKI包括埃克替尼、吉非替尼和厄洛替尼。埃克替尼(商品名：凯美纳)是我国第一个拥有自主知识产权的EGFR-TKI，也是全球第三个上市的EGFR-TKI。自2011年6月7日在中国上市以来已经在临床上积累了50, 000多例NSCLC患者的应用经验。为进一步规范、指导临床医生更好的使用埃克替尼，更好地为肺癌患者服务，中国医师协会肿瘤医师分会和中国抗癌协会肿瘤临床化疗专业委员会组织全国专家，结合我国多个肺癌诊疗规范和指南制定本专家共识。

## 1 *EGFR*基因敏感突变晚期NSCLC患者的一线治疗

大量研究表明，*EGFR*基因突变状态是晚期NSCLC最重要的疗效预测因子和选择治疗方式的分子指标。突变最常见于18-21外显子，其中19外显子缺失突变和21外显子点突变是最常见的*EGFR*基因敏感突变。肺癌突变联盟(Lung Cancer Mutation Consortium, LCMC)的最新研究发现，*EGFR*基因敏感突变的晚期NSCLC患者接受EGFR-TKI治疗后的中位生存时间可达到4年^[2]^。多项研究结果也显示，非选择的中国NSCLC患者*EGFR*基因敏感突变率约为30%，而肺腺癌患者的敏感突变率可以达到50%^[3]^，不吸烟的腺癌患者甚至可高达60%-70%，肺鳞癌患者的*EGFR*突变率为10%左右^[4, 5]^，因此对临床上已经明确NSCLC的病理诊断、并且不能进行手术的晚期患者，在治疗开始前应常规进行*EGFR*基因突变检测。近年来多项一线随机Ⅲ期临床研究(包括IPASS、NEJ002、WJTOG3405、OPTIMAL、EURTAC、LUXLUNG3、LUXLUNG6)^[6-12]^的结果显示，EGFR-TKI一线治疗*EGFR*基因敏感突变晚期NSCLC患者的无进展生存期(progression free survival, PFS)可以达到9.5个月-13.7个月，远高于传统一线化疗方案的4.6个月-6.9个月，总体有效率也同样显示EGFR-TKI明显优于传统化疗(EGFR-TKI和传统化疗分别为58%-84%和15%-47%)。并且所有研究均显示EGFR-TKI的不良反应更轻，尤其是血液学毒性，患者耐受性更好，生活质量明显改善。埃克替尼上市后开展了Ⅳ期临床研究^[13]^，2011年8月至2012年8月共入组了6, 087例接受埃克替尼治疗的晚期NSCLC患者，其中989例患者接受了*EGFR*基因突变检测，738例*EGFR*基因敏感突变患者的客观缓解率(objective response rate, ORR)和疾病控制率(disease control rate, DCR)分别是49.2%和92.3%。其中接受埃克替尼一线治疗的患者144例，ORR和DCR分别是56.3%和95.1%。另一项研究^[14]^回顾性分析了2009年3月至2012年1月首都医科大学附属北京胸科医院收治的59例接受埃克替尼治疗的晚期NSCLC患者的疗效，其中20例患者接受埃克替尼一线治疗，部分缓解(partial response, PR)8例，疾病稳定(stable disease, SD)7例，疾病进展(progressive disease, PD)5例。20例埃克替尼一线治疗患者中有8例存在*EGFR*基因敏感突变，19外显子缺失突变的5例患者均获得PR，21外显子点突变患者3例，其中PR 1例、SD 1例、PD 1例。因此2013年和2014年《MIMS恶性肿瘤用药指南》^[15]^以及《中国原发性肺癌诊疗规范(2015年版)》^[16]^均推荐埃克替尼作为*EGFR*基因敏感突变晚期NSCLC患者的一线治疗药物。目前埃克替尼正在开展多项针对*EGFR*基因敏感突变晚期NSCLC患者一线治疗的临床研究，包括一线与化疗对比的注册临床试验CONVINCE研究(NCT01719536)，一线治疗脑转移的BRAIN研究(NCT01724801)和一线治疗老年*EGFR*基因敏感突变患者的研究(NCT01646450)等。2014年11月13日埃克替尼获得中国国家食品药品监督管理总局(China Food and Drug Administration, CFDA)批准用于一线治疗*EGFR*基因敏感突变晚期NSCLC适应症[批件号：2014B02155]，这也是继吉非替尼之后第二个在中国获得该适应症的EGFR-TKI。

## 2 晚期NSCLC的维持治疗

全球多项一线化疗后EGFR-TKI维持治疗研究的结果均显示，*EGFR*基因敏感突变的晚期NSCLC患者可以从EGFR-TKI维持治疗中获益^[17-19]^。一项2009年3月至2012年1月首都医科大学附属北京胸科医院收治的59例接受埃克替尼治疗的晚期NSCLC患者的回顾性研究^[14]^中有2例*EGFR*基因敏感突变患者在一线化疗后接受了埃克替尼维持治疗，疗效均达PR。埃克替尼目前虽然没有维持治疗的前瞻性研究，但是可以进行这方面的探索。

## 3 晚期NSCLC二、三线治疗

ISEL、INTEREST、TITAN、BR21等多项临床研究以及荟萃(*meta*)分析^[20-24]^的结果均显示，对于未经选择的亚裔复发晚期NSCLC患者，EGFR-TKI能明显降低疾病进展风险，提高肿瘤的客观缓解率，总体疗效与标准的二线化疗相当，但耐受性更好。因此奠定了EGFR-TKI在晚期NSCLC二、三线治疗中的地位。ICOGEN研究^[25]^是我国开展的一项非劣效性Ⅲ期临床试验，比较了埃克替尼与吉非替尼二、三线治疗未经选择的晚期NSCLC患者的疗效和安全性。该研究也是全球第一项两个EGFR-TKI之间头对头比较的Ⅲ期临床研究。结果显示埃克替尼的疗效不劣于吉非替尼，主要终点指标PFS埃克替尼组4.6个月、吉非替尼组3.4个月；药物相关不良事件发生率埃克替尼组61%、吉非替尼组70%(*P*=0.046)，常见不良反应腹泻的发生率埃克替尼组较吉非替尼组显著降低(埃克替尼组19%、吉非替尼组28%，P=0.033)。研究中对能收集到肺癌组织标本的患者进行了*EGFR*基因敏感突变状态的检测，发现无论是*EGFR*基因敏感突变型或野生型患者，吉非替尼与埃克替尼的PFS、OS均无差别，PFS：埃克替尼组7.8个月、吉非替尼组5.3个月，OS：埃克替尼组20.9个月、吉非替尼组20.2个月；吉非替尼与埃克替尼对*EGFR*基因敏感突变型患者的PFS、OS均优于野生型患者(*P* < 0.001)。鉴于ICOGEN研究的结果，埃克替尼被CFDA于2011年6月7日批准上市。在2013年和2014年《MIMS恶性肿瘤用药指南》^[15]^、2014年版《临床路径治疗药物释义・肿瘤疾病分册》^[26]^、2015年版《临床路径释义・肿瘤疾病分册》^[27]^、2013年和2014年版《中国表皮生长因子受体基因敏感突变和间变淋巴瘤激酶融合基因阳性非小细胞肺癌诊断治疗指南》^[28]^以及《中国原发性肺癌诊疗规范(2015年版)》^[16]^均推荐埃克替尼用于晚期NSCLC患者的二、三线治疗。

## 4 EGFR-TKI新辅助和辅助治疗

EGFR-TKI的新辅助和辅助治疗目前国际上没有明确的结论，埃克替尼的多项研究正在进行中(NCT02125240, NCT01929200, NCT01843647)。这些研究结果将回答埃克替尼是否可以使*EGFR*基因敏感突变的Ⅱ期-Ⅲa期肺腺癌患者从治疗中获益。

## 5 小结

总之，埃克替尼使我国有了国产的EGFR-TKI，使我国NSCLC患者有了新的治疗选择，专家委员会将随着研究结果的不断增多，适时更新本共识。

**Table:** 

**专家委员会**	
**顾问**	
**孙燕**	**中国医学科学院北京协和医学院肿瘤医院，抗肿瘤分子靶向药物临床研究北京市重点实验室**
**主任委员**	
**石远凯**	**中国医学科学院北京协和医学院肿瘤医院，抗肿瘤分子靶向药物临床研究北京市重点实验室**
**委员(排名不分先后，按姓氏笔画为序)**	
**丁翠敏**	**河北医科大学第四医院**
**王子平**	**中国医学科学院北京协和医学院肿瘤医院，抗肿瘤分子靶向药物临床研究北京市重点实验室**
**王正**	**深圳市人民医院**
**王长利**	**天津医科大学肿瘤医院**
**石远凯**	**中国医学科学院北京协和医学院肿瘤医院，抗肿瘤分子靶向药物临床研究北京市重点实验室**
**白冲**	**第二军医大学附属长海医院**
**白春学**	**复旦大学附属中山医院**
**冯继锋**	**江苏省肿瘤医院**
**刘晓晴**	**解放军307医院**
**孙燕**	**中国医学科学院北京协和医学院肿瘤医院，抗肿瘤分子靶向药物临床研究北京市重点实验室**
**李方**	**中国人民解放军总医院**
**杨跃**	**北京肿瘤医院**
**束永前**	**江苏省人民医院**
**吴密璐**	**青海大学附属医院**
**何建行**	**广州医科大学附属第一医院**
**张沂平**	**浙江省肿瘤医院**
**张树才**	**首都医科大学附属北京胸科医院**
**陈公琰**	**哈尔滨医科大学附属肿瘤医院**
**罗红鹤**	**中山大学附属第一医院**
**罗荣城**	**南方医科大学南方医院**
**周彩存**	**同济大学附属上海市肺科医院**
**周燕斌**	**中山大学附属第一医院**
**庞青松**	**天津医科大学肿瘤医院**
**赵宏**	**中国人民解放军总医院**
**赵琼**	**浙江大学附属第一医院**
**顾爱琴**	**上海交通大学附属胸科医院**
**凌扬**	**常州市第四人民医院**
**黄诚**	**福建省肿瘤医院**
**韩宝惠**	**上海交通大学附属胸科医院**
**焦顺昌**	**中国人民解放军总医院**
**简红**	**上海交通大学附属胸科医院**

## References

[1] Chen WQ, Zheng RS, Zeng HM (2015). Annual report on status of cancer in China, 2011. Chin J Cancer Res.

[b2] 2Mark G.Kris, Bruce Johnson, Lynne Berry, *et al*.Treatment with therapies matched to oncogenic drivers improves survival in patients with lung cancers: results from the lung cancer mutation consortium (LCMC).15th World Conference on Lung Cancer (WCLC): Abstract PL0307. Presented October 29, 2013.

[3] Shi Y, Au JS, Thongprasert S (2014). A prospective, molecular epidemiology study of *EGFR* mutations in Asian patients with advanced non-small-cell lung cancer of adenocarcinoma histology (PIONEER). J Thorac Oncol.

[4] Wu YL, Zhong WZ, Li LY (2007). Epidermal growth factor receptor mutations and their correlation with gefitinib therapy in patients with non-small cell lung cancer: a *meta*-analysis based on updated individual patient data from six medical centers in mainland China. J Thorac Oncol.

[5] China experts committee for non-small cell lung cancer patients with epidermal growth factor receptor gene mutation detection, Liang ZY (2011). China experts committee consensus on non-small cell lung cancer patients with epidermal growth factor receptor gene mutation detection. Zhonghua Bing Li Xue Za Zhi.

[6] Mok TS, Wu YL, Thongprasert S (2009). Gefitinib or carboplatin-paclitaxel in pulmonary adenocarcinoma. N Engl J Med.

[7] Maemondo M, Inoue A, Kobayashi K (2010). Gefitinib or chemotherapy for non-small-cell lung cancer with mutated *EGFR*. N Engl J Med.

[8] Mitsudomi T, Morita S, Yatabe Y (2010). Gefitinib versus cisplatin plus docetaxel in patients with non-small-cell lung cancer harbouring mutations of the epidermal growth factor receptor (WJTOG3405): an open label, randomised phase 3 trial. Lancet Oncol.

[9] Zhou C, Wu YL, Chen G (2011). Erlotinib versus chemotherapy as first line treatment for patients with advanced *EGFR* mutation positive non small cell lung cancer (OPTIMAL, CTONG-0802): a multicentre, open-label, randomized, phase 3 study. Lancet Oncol.

[10] Rosell R, Carcereny E, Gervais R (2012). Erlotinib versus standard chemotherapy as first-line treatment for European patients with advanced *EGFR* mutation-positive non-small-cell lung cancer (EURTAC): a multicentre, open-label, randomized, phase 3 trial. Lancet Oncol.

[11] Sequist LV, Yang JC, Yamanoto N (2013). Phase Ⅲ study of afatinib or cisplatin plus pemetrexed in patients with metastatic lung adenocarcinoma with *EGFR* mutation. J Clin Oncol.

[b12] 12Wu YL, Zhou CC, Hu CP, *et al*. LUX-Lung6: A randomized, open label, phase Ⅲ study of afatinib (A) versus gemcitabine/cisplatin (GC) as first line treatment for Asian patients with *EGFR* mutation positive advanced adenocarcinoma of the lung. J Clin Oncol, 2013, 31: Abstract8016.

[13] Hu X, Han B, Gu A (2014). A single-arm, multicenter, safety-monitoring, phase Ⅳ study of icotinib in treating advanced non-small cell lung cancer (NSCLC). Lung Cancer.

[14] Li X, Yang XJ, Sun YF (2012). Clinical observation of icotinib hydrochloride for patients with advanced non-small cell lung cancer. Zhonghua Zhong Liu Za Zhi.

[b15] 15黄慧萍, 林慧仪, 主编. MIMS oncology guide恶性肿瘤用药指南. 香港: 美迪医讯亚太有限公司, 2014: 12.

[16] Zhi XY, Shi YK, Yu JM (2015). Standards for the diagnosis and treatment of primary lung cancer in China (2015 version). Zhonghua Zhong Liu Za Zhi.

[17] Zhang L, Ma S, Song X (2012). Gefitinib versus placebo as maintenance therapy in patients with locally advanced or metastatic non small cell lung cancer (INFORM, CTONG0804): a multicentre, double blind randomized phase 3 trial. Lancet Oncol.

[18] Takeda K, Hida T, Sato T (2010). Randomized phase Ⅲ trial of platinum doublet chemotherapy followed by gefitinib compared with continued platinum doublet chemotherapy in Japanese patients with advanced non small cell lung cancer: results of a West Japan Thoracic Oncology Group trial (WJTOG0203). J Clin Oncol.

[19] Cappuzzo F, Ciuleanu T, Stelmakh L (2010). Erlotinib as maintenance treatment in advanced non small cell lung cancer: a multicentre, randomized, placebo controlled phase 3 study. Lancet Oncol.

[20] Thatcher N, Chang A, Parikh P (2005). Gefitinib plus best supportive care in previously treated patients with refractory advanced non-small-cell lung cancer: results from a randomised, placebo-controlled, multicentre study (Iressa Survival Evaluation in Lung Cancer). Lancet.

[21] Kim ES, Hirsh V, Mok T (2008). Gefitinib versus docetaxel in previously treated non-small-cell lung cancer (INTEREST): a randomised phase Ⅲ trial. Lancet.

[22] Ciuleanu T, Stelmakh L, Cicenas S (2012). Efficacy and safety of erlotinib versus chemotherapy in second-line treatment of patients with advanced, non-small-cell lung cancer with poor prognosis (TITAN): a randomised multicentre, open-label, phase 3 study. Lancet Oncol.

[23] Shepherd FA, Rodriques Pereira J (2005). Erlotinib in previously treated non-small-cell lung cancer. N Engl J Med.

[b24] 24Shepherd FA, Douillard J, Fulcuolca M, et al. Comparison of gefitinib and docetaxel in patients with pretreated advanced non-small cell lung cancer (NSCLC): *Meta*-analysis from four clinical trials. J Clin Oncol, 2009, 27(Abstract8011).

[25] Shi YK, Zhang L, Liu XQ (2013). Icotinib versus gefitinib in previously treated advanced non-small-cell lung cancer (ICOGEN): a randomized, double-blind phase 3 non-inferiority trial. Lancet Oncol.

[b26] 26顾晋, 石远凯, 孙忠实, 主编. 临床路径治疗药物释义·肿瘤疾病分册. 北京: 中国协和医科大学出版社, 2014: 334.

[b27] 27石远凯, 顾晋, 主编. 临床路径释义·肿瘤疾病分册(下). 北京: 中国协和医科大学出版社, 2015: 1-38.

[28] Chinese Association for Clinical Oncologists, The Council of Cancer Chemotherapy of Chinese Anti-Cancer Association (2014). The diagnosis and treatment guideline of Chinese patients with *EGFR* gene active mutation and *ALK* fusion gene-positive non-small cell lung cancer (2014 version). Zhonghua Zhong Liu Za Zhi.

